# A CRISPR/Cas9 approach reveals that the polymerase activity of DNA polymerase β is dispensable for HIV-1 infection in dividing and nondividing cells

**DOI:** 10.1074/jbc.M117.793661

**Published:** 2017-07-06

**Authors:** Russell W. Goetze, Dong-Hyun Kim, Raymond F. Schinazi, Baek Kim

**Affiliations:** From the ‡ Department of Pediatrics, Center for Drug Discovery, Emory University, Atlanta, Georgia 30322,; the § School of Pharmacy, Kyung-Hee University, 2447 Seoul, South Korea, and; the ¶ Children's Healthcare of Atlanta, Atlanta, Georgia 30329

**Keywords:** CRISPR/Cas, DNA repair, human immunodeficiency virus (HIV), macrophage, nucleotide, DNA polymerase β, integration

## Abstract

Retrovirus integration into the host genome relies on several host enzymes, potentially including DNA polymerase β (Pol β). However, whether human Pol β is essential for lentivirus replication in human cells is unclear. Here, we abolished DNA polymerase β (Pol β) expression by targeting its DNA polymerase domain with CRISPR/Cas9 in human monocytic THP-1 cells to investigate the role of Pol β in HIV-1 transduction in both dividing and nondividing macrophage stages of THP-1 cells. Pol β–knock-out was confirmed by enhanced sensitivity to methyl methanesulfonate-induced DNA damage. Of note, nuclear extracts from Pol β–knock-out THP-1 cells prepared from both dividing and nondividing stages displayed significantly reduced capability to repair the gapped HIV-1 integration intermediate DNA substrate in a biochemical simulation. However, nuclear extract from both dividing and nondividing stages of the Pol β–KO cells had detectable gap repair activity, suggesting that other host DNA polymerases also repair gapped HIV-1 DNA, particularly in dividing cells. Next, when we compared transduction using HIV-1 and simian immunodeficiency virus in control and Pol β–KO cells, the loss of the Pol β expression did not affect transduction efficiency of these lentiviruses in both dividing and nondividing stages. Finally, the gap repair assay indicated that limited cellular dNTP pools, but not Pol β expression, are a primary factor for HIV-1 DNA gap repair, particularly in nondividing cells. These data support the idea that Pol β polymerase activity is dispensable for HIV-1 infection in both dividing and nondividing stages of human cells targeted by the virus.

## Introduction

One of the hallmarks of retrovirus replication is integration of viral DNA into a host chromosome of infected cells. Integration of lentiviruses such as human immunodeficiency virus Type 1 (HIV-1) and simian immunodeficiency virus (SIV)^2^ requires highly coordinated actions of both viral and host players. After the viral double-stranded (ds) DNA is synthesized from the viral genomic RNA by reverse transcriptase (RT), a number of viral and host proteins coordinate to assemble the pre-integration complex, which transports the viral dsDNA into the nucleus where it is inserted into a host chromosome (1). The integration process consists of three distinct and sequential steps: 1) 2–3 nucleotides are removed from both 3′ ends of the viral dsDNA by the viral integrase (IN); 2) the 3′ ends of the viral dsDNA are covalently linked to the chromosomal DNA of the host by transesterification catalyzed by IN (2); and 3) a 4–6 nucleotide single-stranded (ss) DNA gap between the 5′ end of the viral DNA and 3′ end of the host chromosomal DNA is filled and ligated after removing mismatches at the 5′ ends of the viral DNA (3), resulting in a completely integrated provirus. Although the viral IN catalyzes the first two steps, the third step is thought to be largely carried out by the host DNA repair machinery (4).

Among the host proteins involved in the DNA repair process, DNA polymerase β (Pol β) is speculated to be the enzyme responsible for filling the ssDNA gap resulting from viral integration (4). Pol β is known to act in base excision repair (BER), which repairs DNA damage resulting from sources such as alkylating agents and reactive oxygen species (5). Aberrations in Pol β expression and activity have been reported in various cancers (6–8). Recently, a study reported that Pol β knockdown by RNAi in HeLa cells reduces HIV-1 transduction in a targeted screen of DNA repair enzymes (9). Additionally, reduction in HIV-1 and FIV infectivity were reported in mouse embryonic fibroblasts from *POLB*^−/−^ animals (10). Results from these studies support the role of Pol β and other BER enzymes in lentivirus integration. Our laboratory has also reported that immunodepletion of Pol β from primary CD4+ T cell and macrophage nuclear extract reduces HIV-1 ssDNA gap repair activity biochemically (11). However, genetic evidence clarifying the role of human Pol β in lentivirus replication in human cells remains to be reported.

Another unique feature of lentiviruses relative to other members of the *Retroviridae* family is the ability to replicate in both dividing and nondividing cells (12). In the case of HIV-1 and SIV, activated CD4+ T cells and macrophages, respectively, represent important targets of infection within this classification. Because nondividing cells lack chromosomal DNA synthesis, it is plausible that the DNA repair mechanisms used by lentiviruses during integration may be regulated differently between these two cell types. In fact, to address questions relating to dividing and nondividing target cells, the THP-1 cell model, a monocytic leukemia cell line, has been extensively used because dividing THP-1 cells can be differentiated to a nondividing macrophage-like phenotype by treatment with phorbol 12-myristate 13-acetate (PMA) (13, 14).

In the present study, we generated novel *POLB* KO THP-1 cell lines using a CRISPR/Cas9 system (15). These KO cell lines were validated and shown to both display enhanced sensitivity to alkylating agents and to lack efficient ssDNA gap repair activity *in vitro*. Unlike previous reports, which showed more pronounced reductions in viral transduction efficiency in Pol β knockdown human cells and mouse knock-out cells (9, 10), we observed only minor, yet statistically significant, effects of the loss of Pol β on HIV-1 and SIV transduction efficiency in both dividing and nondividing *POLB* KO THP-1 cells. Furthermore, we show that the rate of ssDNA gap repair is limited at physiological dNTP concentrations, which are further restricted in nondividing cells. Our results suggest that Pol β is not essential to the ssDNA gap repair during lentiviral transduction in both dividing and nondividing cells. Additionally, this repair process is kinetically limited by cellular dNTP concentrations particularly in nondividing cells.

## Results

### POLB KO in THP-1 cells using CRISPR/Cas9-based gene editing

**Figure 1.**
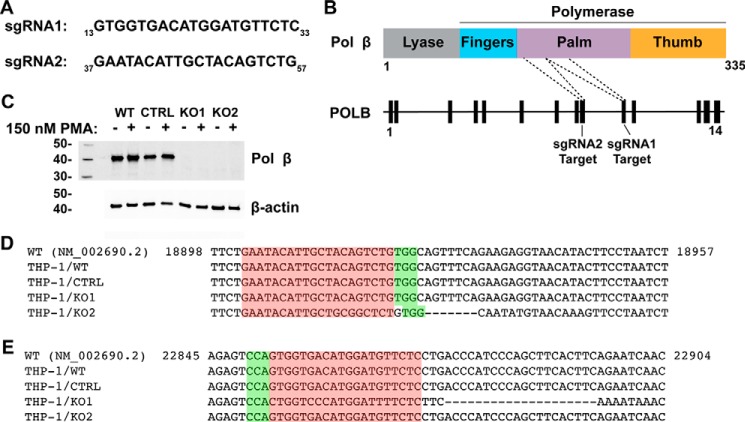
**Generation of *POLB* KO THP-1 cell lines by CRISPR/Cas9.** *A,* sgRNA sequences used in this study. The nucleotide numbers within exon 10 (sgRNA1) or exon 9 (sgRNA2) of the *POLB* gene are indicated as a *subscript. B,* map of the Pol β protein and *POLB* gene. sgRNA1 and sgRNA2 target regions within exon 10 and 9, respectively. Both targets are within a coding region that corresponds to the palm subdomain of the DNA polymerase domain. Amino acid numbering and subdomains of the Pol β protein are indicated. Exon numbers are indicated for the *POLB* gene. *C,* nuclear extracts were isolated from the dividing (−*PMA*) or nondividing macrophage (+*PMA*) stages of THP-1 cells. Nuclear extracts (10 μg/lane) from wild-type (*WT*) THP-1, empty vector control (*CTRL*), and *POLB* KO THP-1 cells (*KO1* and *KO2*) were probed with a monoclonal anti-Pol β antibody targeting the C-terminal region. Blots were stripped and re-probed for β-actin as a loading control. Positions of molecular weight markers are indicated on the *left side* of the blot. Results are representative of two independent experiments. Genomic DNAs from WT THP-1, CTRL, KO1, and KO2 cells were isolated and PCR amplicons flanking the CRISPR/Cas9-targeted regions were sequenced. Sequence alignments of bases 18898–18957 (exon 9) (*D*) and 22845–22904 (exon 10) (*E*) of the *POLB* gene are shown. Numbering is based on the entire *POLB* gene sequence using the reference gene RefSeq NM_002690.2.

Previously reported (10) cellular *POLB* KO models used to study HIV-1 replication are derived from mice, which may not faithfully recapitulate the normal host environment of primate lentiviruses. Also, only RNAi-based tests have been used to study the role of human Pol β in HIV integration (9). To generate a novel and relevant human cellular model, we employed LentiCRISPRv2 (15), a lentiviral vector-based CRISPR/Cas9 delivery system expressing target sgRNA, Cas9 nuclease, and a puromycin selection marker to induce *POLB* deletion. We selected single guide RNA (sgRNA) sequences (Fig. 1*A*) from the Genome-scale CRISPR KO (GeCKO) database (16) targeting two different regions near the polymerase active site of the Pol β palm subdomain (Fig. 1*B*). More specifically, sgRNA1 targets exon 10 of the *POLB* gene, a region within the highly structured palm domain, which encodes the metal binding triad, dNTP-binding site, primer-binding site, and active site. sgRNA2 targets exon 9 and corresponds to a structured region in the palm domain proximal to the active site, but does not directly encode any catalytic residues.

Next, we chose the human monocytic THP-1 cell line for *POLB* KO because this cell line is both able to be efficiently infected by HIV-1 and can be differentiated to a nondividing macrophage stage by treatment with PMA. THP-1 cells were transduced with each of the constructed lentiviral vectors including the empty vector as a control. Following transduction, puromycin selection, and single-cell sorting, clonal cells were expanded and assessed for successful *POLB* KO by Western blot analysis in both dividing (−PMA) and nondividing, differentiated stages (+PMA) (Fig. 1*C*). We isolated genomic DNAs from Western blot hit clones, sequenced exons 9 (Fig. 1*D*) and 10 (Fig. 1*E*) of *POLB*, and then chose one clone corresponding to each sgRNA for further characterization. We found that the clone edited with sgRNA1 (KO1) had several point mutations near the target site, which induce amino acid changes and a 7-base pair frameshifting deletion that introduces a premature stop codon downstream ∼50 base pairs of the deletion. The clone edited with sgRNA2 (KO2) also had several point mutations near the target sequence and a 20-base pair frameshifting deletion that introduces a premature stop codon ∼100 base pairs downstream. Additionally, clonal THP-1 cells transduced with the LentiCRISPRv2 transfer vector lacking a sgRNA insert (empty vector) were selected for use as a control that expresses Pol β in both dividing and nondividing stages, consistent with what we observed in the parental THP-1 cells (Fig. 1*C*). These data provide sufficient molecular and genetic evidence that the selected clones were *POLB* null to proceed with further functional analysis.

### POLB KO THP-1 cells are sensitive to methyl methanesulfonate (MMS)-induced DNA damage

**Figure 2.**
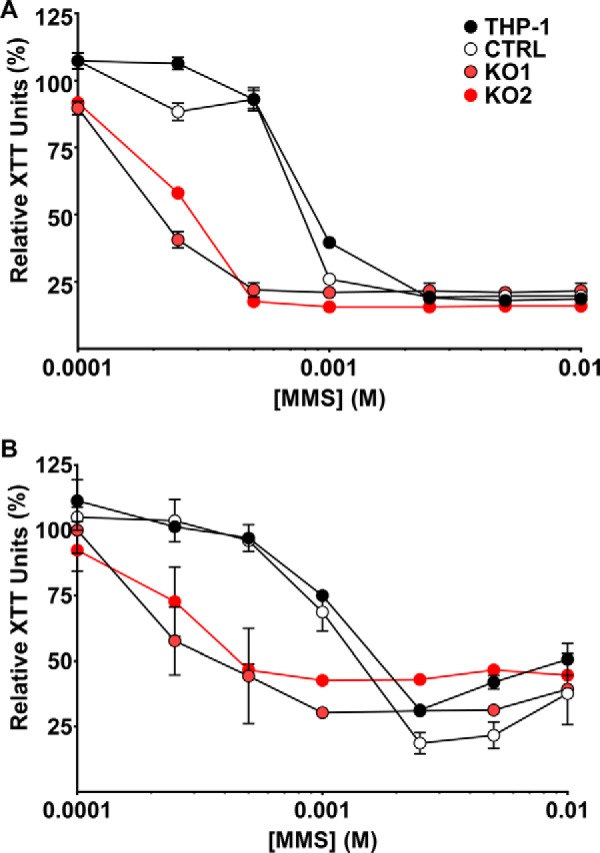
**POLB KO THP-1 cells are sensitive to MMS-induced DNA damage.** 2.5 × 10^4^ THP-1 cells per well were seeded in 96-well plates in the dividing (*A*) or nondividing (*B*) stage. WT THP-1, CTRL, KO1, and KO2 cells were treated with varying concentrations of the DNA alkylating agent MMS for 2 h. Cells were washed three times with 1× PBS and cultured for 72 h. Cell viability was measured using the XTT assay and values were normalized to untreated control for each group. Results from two independent experiments performed in triplicate are shown as mean ± S.D.

*POLB* KO has been validated in previous cellular systems using sensitivity to MMS (5, 17), which induces DNA damage repaired by the BER pathway. We treated our THP-1 clones as well as the polyclonal parental THP-1 cells with a range of MMS concentrations and evaluated cell sensitivity to MMS using an XTT-based cell proliferation assay. *POLB* KO cell lines, but not empty vector control cells, showed an enhanced susceptibility to MMS (Fig. 2*A*) that was consistent with previously published values using growth inhibition assays (5, 17).

Additionally, we stimulated THP-1 cells with PMA to induce a macrophage-like phenotype as previously described (13). In this state, THP-1 cells have decreased dNTP concentrations (18) and are nondividing with little cellular DNA synthesis (supplemental Fig. S1). Next, we tested whether nondividing THP-1 cells were also sensitive to DNA damage by MMS. Nondividing KO1 and KO2 cells showed sensitivity to MMS-induced DNA damage compared with parental THP-1 and CTRL cells (Fig. 2*B*) similar to the results observed in dividing THP-1 cells. Based on these functional results, we concluded that the cellular phenotype presented by our *POLB* KO cells was consistent with previously reported findings and supports the validity of our molecular analysis.

### POLB KO THP-1 nuclear extracts have impaired HIV-1 ssDNA gap repair activity in vitro

**Figure 3.**
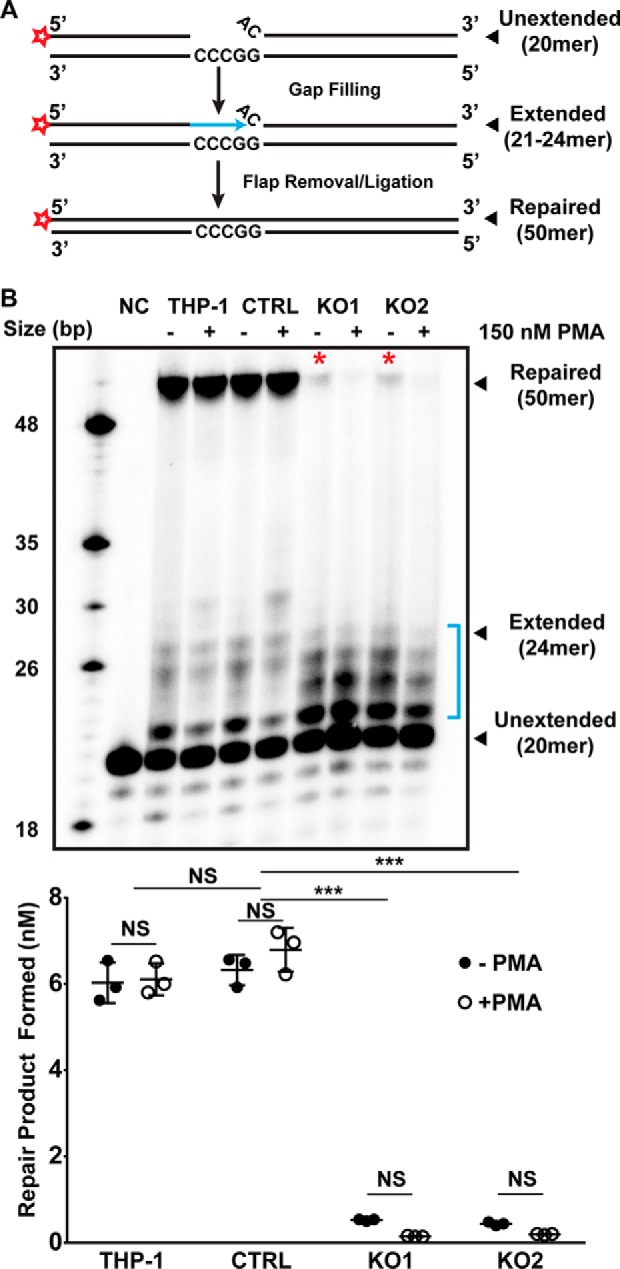
**Effect of *POLB* KO on biochemical ssDNA gap repair activity with HIV-1 gap DNA substrate.** Lentiviral gap filling was modeled *in vitro* using a previously reported assay (13). *A,* schematic showing the steps required for complete repair of a model substrate based on the HIV-1 LTR. A 5′ end ^32^P-labeled 20-mer oligonucleotide primer (*red stars*) and an unlabeled 27-mer oligonucleotide primer with a single 5′ end mismatch are annealed to a 50-mer oligonucleotide template. Extension of the labeled primer by DNA polymerases yields 21–24-mer products (indicated by *blue arrow*), which require mismatch removal and ligation to the unlabeled 27-mer to form the labeled 50-mer gap repair product. *B,* 20 nm gap repair substrate was incubated with 4 μg of nuclear extract from WT THP-1, CTRL, KO1, and KO2 cells in the presence of 250 μm dNTPs and 2 mm ATP. Reactions were incubated at 37 °C for 30 min, then quenched with 40 mm EDTA and inactivated at 95 °C for 1 min. The substrate was also incubated with the WT THP-1 nuclear extract in the absence of dNTPs (*NC*) at 37 °C for 30 min (*NC*). Products were resolved by urea-PAGE on a 20% acrylamide gel and visualized by phosphorimaging. 20-Mer unextended substrate, 24-mer intermediate (including partially extended products indicated by *bracket*), and 50-mer fully repaired product are indicated. The results were quantitated by densitometry in ImageLab 5.2 (Bio-Rad). The amount of repair product was calculated as ratio of 50-mer band density to total density per lane and related to the concentration of substrate in each reaction. Results from three independent experiments are shown as mean ± S.D. Two-way analysis of variance was performed and Dunnett's multiple comparison test was used to determine differences between cell lines and the effect of PMA treatment. ***, *p* < 0.001. *NS*, not significant.

We previously reported an *in vitro* assay that simulates the single-strand DNA gap repair mechanism that occurs during the integration step of HIV-1 by using nuclear extracts (11). This system requires three enzymatic steps to occur to generate a 50-mer gap repair product as illustrated in Fig. 3*A*: 1) DNA synthesis from the ^32^P-labled 5′-end of a DNA primer across a four-nucleotide gap generated from the annealed 3′ DNA primer; 2) displacement and excision of a mismatched single-nucleotide flap; and 3) ligation of the extended 5′ and 3′ primers, generating the 50-mer repaired product.

To test whether gap repair was restricted in *POLB* KO cells, we prepared nuclear extracts from parental THP-1, empty vector control, KO1, and KO2 cells in both dividing (−PMA) and nondividing (+PMA) stages as previously described (11). Nuclear extracts were normalized to 1 mg/ml of total protein by the Bradford assay and incubated with the radiolabeled gap repair substrate described in the legend to Fig. 3*A* with saturating dNTPs (250 μm) for 30 min at 37 °C. As shown in Fig. 3*B*, *POLB* KO1 and KO2 nuclear extracts generated significantly decreased 50-mer repaired product, compared with both parental and control THP-1 cells expressing Pol β. Surprisingly, however, the *POLB* KO nuclear extracts still displayed some levels of the partially extended 5′ primer (see “*bracket*” in Fig. 3*B*), supporting that other DNA polymerases can recognize the HIV-1 gap substrate, whereas Pol β appears to be the primary polymerase that recognizes the substrate. Also, when the repaired product in each reaction was quantitated (Fig. 3*C*), no significant difference in the DNA gap repair activity between dividing (−PMA) and nondividing (+PMA) stages of all cell types, suggesting that Pol β is also the major polymerase for the ssDNA gap repair in the nondividing stage. Furthermore, this observation is supported by the increased sensitivity to MMS we observed in the PMA-treated *POLB* KO cells (Fig. 2*B*). Taken together, these data suggest that the genetic loss of Pol β expression significantly reduces, but not completely abolish the HIV-1 ssDNA gap repair activity in the THP-1 model.

### POLB KO has limited effects on lentivirus transduction in both dividing and nondividing THP-1 cells

**Figure 4.**
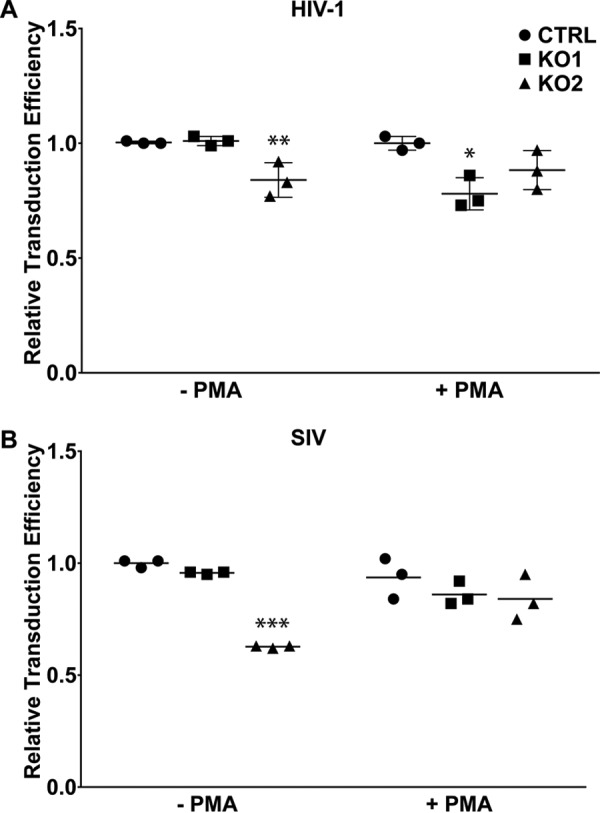
**Effect of *POLB* KO on HIV-1 transduction in dividing and nondividing THP-1 cells.** CTRL, KO1, and KO2 cells were grown in suspension culture for the dividing stage (−*PMA*) or treated with 150 nm PMA (+*PMA*) for 7 days to differentiate into the nondividing macrophage stage. Cells were transduced with VSV-G-pseudotyped HIV-1 (*A*) or SIV_mac239_ (*B*) vector expressing GFP at an multiplicity of infection of 0.4 and assayed by flow cytometry to measure the GFP expressing population at 24 (dividing) or 120 h (nondividing) post-transduction. Data are reported as mean ± S.D. of three independent experiments performed in triplicate. Data were analyzed by one-way analysis of variance and differences between KO and CTRL cells were determined using Dunnett's multiple comparison test. *, *p* < 0.05. **, *p* < 0.01. ***, *p* < 0.001.

A previous report showed that embryonic fibroblasts derived from *POLB*^−/−^ mice exhibited reduced lentivirus transduction compared with wild-type cells (10). However, this model is not completely relevant because mice do not carry lentiviruses. Therefore, we employed our human *POLB* KO THP-1 cells to test whether human Pol β is involved in HIV-1 transduction. For this test, THP-1 empty vector, KO1, and KO2 cells were infected in dividing and nondividing stages with VSV-G pseudotyped GFP-reporter HIV-1 (DHIV3-GFP) pseudovirus that encodes all of the NL4-3 genes except *env* and *nef*, which are replaced with GFP (19). As shown in Fig. 4*A*, only minor differences in HIV-1 vector transduction efficiency relative to empty vector control cells was observed in both dividing and nondividing stages of THP-1 cells. We found that transduction efficiency was reduced by ∼20% in both dividing KO2 cells and nondividing KO1 cells. We also observed that *POLB* KO did not uniformly reduce the transduction efficiency of another lentivirus, SIV (Fig. 4*B*). The absence of a uniform effect in these data suggest that human Pol β is not absolutely required for HIV-1 transduction. This result is further supported by the biochemical data presented in Fig. 3 that other DNA polymerases may also recognize the HIV-1 ssDNA gap although their capability to repair the HIV-1 gap is less efficient than Pol β.

### In vitro HIV-1 ssDNA gap repair is dependent on dNTP concentration

**Figure 5.**
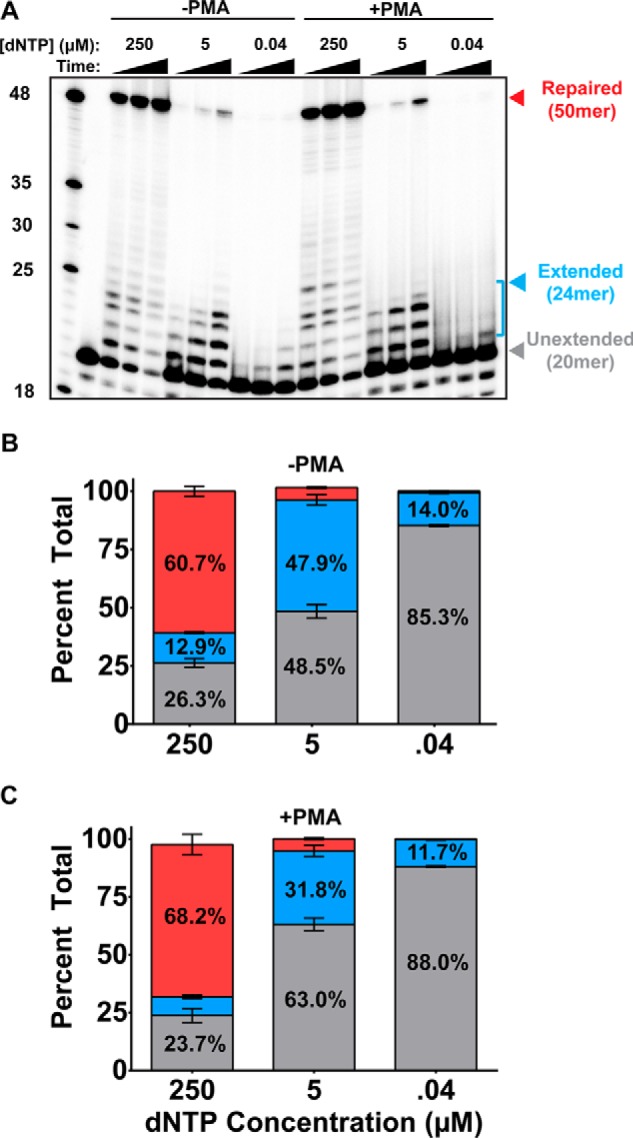
**Effect of dNTP concentration on *in vitro* HIV-1 ssDNA gap repair activity.** Nuclear extracts from dividing (−*PMA*) and nondividing (+*PMA*) stages of THP-1 CTRL cells expressing Pol β were isolated. *A,* 20 nm gap repair substrate was incubated with 4 μg of nuclear extract from CTRL cells in the presence of saturating (250 μm), dividing cell (5 μm), or nondividing cell (40 nm) concentrations of dNTPs and 2 mm ATP (required for ligation). Reactions were incubated for 30, 60, or 120 min. Data from three independent experiments were analyzed by densitometry to quantitate the amounts of fully repaired, partially extended (*blue bracket*), and unextended radiolabeled primers, which are reported as mean ± S.D. for dividing (*B*) and nondividing (*C*) stages of THP-1 empty vector control cells. The percentage comprised by each product (repaired, partially extended, and unextended) are represented by *red*, *blue*, and *gray bars*, respectively. The mean percentage calculated for each product is indicated by the number inside the corresponding bar.

We previously reported that the cellular dNTP level in nondividing cells such as human monocyte-derived macrophages is extremely low (20–40 nm), compared with dividing cells such as activated CD4+ T cells (1–4 μm) (20). The extremely limited dNTP pool in macrophages kinetically suppresses HIV-1 reverse transcription, which consumes cellular dNTP substrates for viral dsDNA synthesis (21). However, it is also clear that the HIV-1 DNA gap repair during viral integration requires cellular dNTPs. Indeed, our previous *in vitro* HIV-1 DNA gap repair assay demonstrated that the cellular dNTP concentration affects the HIV-1 DNA gap repair (11). Here we tested the effect of the dNTP concentration on the HIV-1 DNA gap repair activity of the nuclear extract prepared from our THP-1 cell line model. For this test, we performed the *in vitro* HIV DNA gap repair assay using nuclear extracts from dividing (−PMA) and nondividing (+PMA) stages of parental THP-1 at dNTP concentrations found in dividing/activated CD4+ T cells (2.5 μm), nondividing macrophages (40 nm), as well as a saturating concentration (250 μm) (Fig. 5). We measured the two product populations: 1) fully repaired product (50-mer product in Fig. 5*A,* and *red portions* in *B* and *C*) and 2) the partially extended product (*bracket* in Fig. 5*A,* and *blue* in *B* and *C*). First, the levels of the fully repaired 50-mer product were significantly reduced at both intracellular dNTP concentrations (2.5 μm and 40 nm), compared with saturating dNTPs (250 μm), although the repair product was still detectable at later time points at the dividing cell dNTP concentration (2.5 μm). However, no fully repaired product was detected at the nondividing cell dNTP concentration (40 nm) even at the later time points. Second, this dNTP concentration-dependent gap repair activity was observed in both PMA-treated and untreated THP-1 cells (Fig. 5*A*). Third, when we quantitated the total primer extension level including both partially extended products and fully repaired 50-mer products, the dividing cell dNTP concentration (2.5 μm) gave higher levels (∼48%) of the total extended product than the macrophage dNTP concentration (∼14%) in reactions with the PMA-untreated cell extract (Fig. 5*B*), and a similar difference in the total extended products was also observed in the reactions with the PMA-treated cells (Fig. 5*C*). This simulation data supports that the availability of intracellular dNTPs significantly affects HIV-1 DNA gap repair and that the limited dNTP pools, not Pol β expression, is a primary limiting factor to control the HIV-1 gap repair in nondividing cells, which at least partially explains the absence of an effect of *POLB* KO on HIV-1 infectivity in the nondividing stage of THP-1 cells (Fig. 4).

## Discussion

Integration of genetic information by lentiviruses such as HIV-1 presents a significant barrier to eradicating the virus *in vivo*. Although all retroviruses encode a viral IN (1), none encode enzymes that process the 5′ ends of the partially integrated viral dsDNA, a substrate that requires the relatively complex removal of mismatched base pairs and repair of the ssDNA gap. This involves multiple enzymatic functions including removal of mismatched bases by flap endonuclease, DNA polymerization, and ligation of the newly synthesized DNA to a host chromosome (3). The provirus is thought to be stably integrated only after these steps have been completed. Because the host DNA repair polymerase Pol β is known to fill short ssDNA gaps during routine cellular DNA repair, it has been speculated that Pol β is involved in lentiviral 5′-end DNA gap repair. This was supported by the findings of a targeted siRNA screen that showed a modest reduction of HIV-1 transduction in HeLa cells when some BER enzymes, including Pol β, were knocked down (9). This work was further supported by findings demonstrating modest reductions in HIV-1 infectivity in embryonic fibroblasts derived from *POLB*^−/−^ mice (10). However, these results have not been confirmed by genetic evidence in human cell models. Importantly, to the best of our knowledge, the present study is the first to report human *POLB* KO cells and therefore provides the most complete system for modeling HIV-1 gap repair in human cells to date.

Lentiviruses such as HIV-1 and SIV infect terminally differentiated, nondividing myeloid cells such as macrophages (22, 23). These cells lack chromosomal DNA replication, cell division, and feature additional mechanisms that present barriers to lentiviral replication. Two such mechanisms involve tight control of dNTP biosynthesis by inhibition of ribonucleotide reductase (24) and activation of dNTP hydrolysis by sterile α motif and HD domain-containing protein 1 (SAMHD1) (25, 26). We have previously shown that by limiting the dNTP concentration, nondividing cells restrict lentivirus replication at both the reverse transcription and integration steps (11). Because most research examining DNA repair has focused on dividing cells, the implication of these unique regulations is not well understood. In fact, it remains unclear whether such terminally differentiated, nondividing cells carry fully functional DNA repair capacity in the absence of chromosomal DNA replication and to what extent this is controlled by cell type. Transcription-coupled DNA repair appears to function in nondividing cells, but has been not been fully characterized (27). Interestingly, because of the tight dNTP regulation that occurs in nondividing cells, the cellular DNA polymerases that act in DNA repair pathways may not operate efficiently. We previously reported that macrophages harbor dNTP concentrations in the 20–50 nm range (20), which is much lower than reported *K_m_* values of any known cellular DNA polymerase (1–100 μm) (28–30). Therefore, it is unclear that the DNA repair machinery in nondividing lentivirus target cells are able to efficiently perform 5′ end gap repair under such restrictive conditions. The evidence that we present here is the first to compare gap repair in both dividing and nondividing cells that completely lack Pol β expression. Our previous findings demonstrated that the rate of gap repair was controlled by the dNTP concentration, but these experiments were performed in cells that expressed Pol β (11). These new data validate the significance of cellular dNTP regulation in determining the rate of lentiviral DNA gap repair.

In the present study, we demonstrate that HIV-1 and SIV replicate with little impairment in *POLB* KO cells under both dividing and nondividing conditions. This finding surprised us, considering that others had observed a reduction in transduction efficiency in other systems using mouse *POLB* KO or human *POLB* KD. This led us to consider two possible scenarios: 1) other DNA polymerases besides Pol β perform 5′ end gap repair of partially integrated viral dsDNA, particularly in dividing cells, or 2) lentiviruses may not require completion of 5′ end gap repair to begin transcription of proviral DNA.

For the first scenario, we considered other non-replicative DNA polymerases. Pol β, Pol λ, and Pol μ are all members of DNA polymerase family X and are involved in DNA repair (31). Although Pol μ appears to function primarily in B-cell maturation in lymphoid tissue, Pol λ is known to act as a back-up in BER reactions using cell extracts (32). However, the extent to which Pol λ can fill this role in nondividing cells is unknown. In contrast, Pol β is constitutively expressed with increases in mRNA expression before and during chromosomal DNA replication (33, 34) and following DNA damage (35). This evidence supports the role of Pol β as the primary repair polymerase in nondividing cells, but does not exclude the possibility that other DNA polymerases can perform the same function in its absence. Indeed, our biochemical simulation assay (Fig. 3*A*) showed that the nuclear extracts of the *POLB* KO cells particularly prepared from the dividing stage displayed detectable HIV-1 DNA gap repair activity, supporting the possibility that other DNA polymerases were able to recognize and repair the HIV-1 gap DNA. Notably, under conditions with saturating dNTPs, the rate of gap repair was reduced by more than 90% in *POLB* KO cells (Fig. 3). This indicates that although other DNA polymerases are able to fill the gap in the absence of Pol β, the repair process is much less efficient. However, when this experiment was repeated using *POLB* KO cells and varying dNTP concentrations, the effect size on repair rate between WT and *POLB* KO cells decreased from 10-fold with saturating dNTPs to less than 2-fold under physiological dNTP concentrations (supplemental Fig. S2). These data help to explain why we observed only small differences in transduction efficiency between WT and *POLB* KO cells (Fig. 4), whereas *in vitro* gap repair assays showed such a strong effect (Fig. 3). Because the HIV-1 DNA gap repair is absolutely dependent on the dNTP concentration (Fig. 5 and supplemental Fig. S2), the extremely limited dNTP pools observed in nondividing cells, not Pol β expression, could be a primary factor to control the HIV-1 DNA gap repair during viral integration in nondividing cells. Notably, the dNTP concentration is modestly elevated in nondividing cells in response to DNA damage by p53-dependent induction of the *p53R2* gene, which encodes an alternative small R2 subunit of ribonucleotide reductase (24, 36). However, induction of *p53R2* requires prolonged exposure to DNA damage and is not triggered by HIV-1 infection (18, 36).

In addition to cellular DNA polymerases, RT is capable of filling the gapped DNA intermediate through its strand displacement activity (4, 37, 38). Gap filling by RT directly would also explain the absence of a strong effect on transduction efficiency in *POLB* KO cells, particularly in nondividing cells where the higher efficiency of the RT at low dNTP concentrations could potentially play an important role. Furthermore, there is evidence that RT and IN directly interact (39), which could support localization of RT to the site of integration thereby bypassing a need for a cellular DNA polymerase. However, it is unclear whether RT activity is maintained after the pre-integration complex centers the nucleus because reverse transcription occurs primarily in the cytoplasm (1). Additionally, gap filling by RT would not circumvent the need for cellular endonuclease and ligase activity, which may then act as the rate-limiting step in repair if gap filling by RT is indeed efficient.

For the second scenario, we speculated that immediate repair of the 5′ end LTR gap may not be necessary for HIV replication, particularly in nondividing cells. DNA damage detected during chromosomal replication leads to activation of DNA damage response mechanisms, which induce cell cycle arrest initially and eventually apoptosis, if necessary (40). However, activated CD4+ T cells infected by HIV-1 undergo cell cycle arrest induced by viral protein R (41) and eventually cell death without resuming cell division. In some infected CD4+ T cells that become quiescent, the immediate need for DNA repair may also be bypassed because these cells are no longer cycling. The same logic follows for macrophages, which are also nondividing. Finally, it is not known whether and how the unrepaired 5′ end gap at the LTR affects HIV-1 transcription. Furthermore, transcriptional machinery assembles at structures located within the LTR (1), which would avoid potentially stalled processivity across the 5′ end gap. Although this scenario is purely speculative, it can be tested using methods to detect the unrepaired 5′ end gap of HIV-1 DNA. However, there is currently no reliable quantitative assay for measuring the partially integrated HIV-1 DNA and we were unable to adapt an existing assay used to detect the gap for Molony murine leukemia virus (42).

In addition to DNA polymerase activity, both Pol β and Pol λ have distinct 5′-2-deoxyribose-5-phosphate lyase activity (43–45). Recently, it was reported that Pol β 5′-2-deoxyribose-5-phosphate lyase activity, but not polymerase activity, is required for efficient HIV transduction in mouse embryonic fibroblasts (46). In our study, we considered that because CRISPR-induced deletions were downstream of the Pol β lyase domain that it was possible that a fragment retaining this activity may still be expressed in our *POLB* KO cells. We probed nuclear extracts from WT and *POLB* KO THP-1 cells using a polyclonal antibody raised against whole Pol β protein and were unable to detect any specific band that might represent a truncated form of Pol β (data not shown). Although this is a caveat that could explain our failure to reproduce a reduction in infectivity, it is important to note that these previously reported results relied on expression of a full-length Pol β construct with point mutations in the polymerase or lyase active sites. Because our *POLB* KO cells lack nearly all of palm domain and all of thumb domain, it is unlikely that such a truncated protein would even retain the ability to interact with DNA even if a functional lyase subdomain was expressed. Future experiments can target the lyase domain of Pol β to generate a complete deletion to conclusively determine whether the Pol β lyase is also dispensable for lentivirus replication.

In conclusion, our genetic biochemical investigations revealed that the polymerase activity of Pol β appears to be dispensable for HIV-1 transduction in both dividing and nondividing THP-1 cells. This study raises new possibilities for consideration in HIV-1 5′ end gap repair: 5′ end gap repair of lentiviral DNA may promiscuously utilize cellular DNA polymerases and/or viral RT during integration or that immediate repair of the 5′ end gap may not be necessary for viral replication.

## Materials and methods

### Cell culture and lentiviral vectors

THP-1 cells (ATCC TIB-202) were cultured in RPMI 1640 medium (Corning) supplemented with 10% FBS and 1% penicillin/streptomycin according to the supplier's recommended subculturing method. THP-1 cells were differentiated by continuous stimulation with 150 nm PMA (Sigma) for 7 days with media replaced every 2 days. All cells were grown at 37 °C, 5% CO_2_. 293FT cells (Invitrogen) were cultured in DMEM (Gibco) supplemented with 10% FBS and 1% penicillin/streptomycin according to the supplier's recommended subculturing method. 293FT cells were transfected to produce lentiviral vector as previously described (21). Briefly, cells were grown to 70% confluence and transfected using (Sigma) polyethylenimine-complexed plasmid DNA. Transfection medium was removed after 8 h and supernatant was collected at 24 and 48 h post-transfection. Supernatant was filtered through a 0.45-μm filter and concentrated using a Beckman Coulter Optima XE90 ultracentrifuge. Concentrated virus pellets were resuspended in 1× Hanks' balanced salt solution (Gibco), aliquotted, and stored at −80 °C. Pseudoviruses were produced in 293FT cells co-transfected with GFP-expressing lentivirus plasmid and pCMV-VSV-G (vesicular stomatitis virus G protein under control of the cytomegalovirus immediate-early enhancer and promoter) (47). HIV-1 pseudovirus was produced using pDHIV3-GFP (NL4–3-based Δ*env*, Δ*nef*) (19). SIV pseudovirus was produced using pSIV-GFP and pSIVΔ*vpx*-GFP (SIV_mac239_-based Δ*env*) (48). Core antigen was measured using p24 and p27 ELISA (Advanced BioScience Laboratories Inc.) and RT activity was measured as previously described (49).

### Lentiviral vector transduction

The pseudovirus vector input was normalized by RT activity, then THP-1 cells were infected in the presence 30 μg/ml of DEAE dextran (Sigma) for 2 h, washed 3 times with 1× PBS (Gibco), and analyzed for GFP expression 24 or 120 h post-transduction for dividing and nondividing cells, respectively. GFP measurement was performed using a MACSQuantVYB flow cytometer (Miltenyi Biotec) and data were analyzed using MACSQuantify software (Miltenyi Biotec).

### Human POLB KO by LentiCRISPRv2

LentiCRISPRv2 plasmids targeting *POLB* were constructed using methods previously described (15, 16). Briefly, complimentary oligonucleotides containing the specific sgRNA sequence and overhangs complementary to overhangs generated by BsmBI digestion of LentiCRISPRv2 were annealed to the BsmBI-digested LentiCRISPRv2 plasmid to generate the functional transfer vector. Undigested LentiCRISPRv2 plasmid lacking a sgRNA sequence was used for pseudovirus production as a control. LentiCRISPRv2 vector was generated as described for HIV-1 and SIV pseudoviruses, except that the psPAX2 packaging plasmid (Addgene) was co-transfected with the LentiCRISPRv2 (15) and pCMV-VSV-G plasmids. THP-1 cells were infected with concentrated LentiCRISPRv2 pseudovirus. 48 h after transduction, media was replaced with complete RPMI containing 1 μg/ml of puromycin (Gibco) and maintained in selection media for 14 days. Selected cells were single-cell sorted into 96-well plates using a BD FACS Aria II Cell Sorter (BD Biosciences). Single cells were expanded and assayed for Pol β expression by Western blot analysis. Cells negative for Pol β by Western blot analysis were further analyzed by PCR amplification of genomic DNA flanking the CRISPR-targeted region. The forward primers 5′-CTTGCCTTGTCAGTAGACAGCA-3′ and 5′-CTCTGTGTTGACTGGGTTGGTC-3′ and the reverse primers 5′-AACTTGGGCAGTTGGGCACAGT-3′ and 5′-CCCGGCCATCTCTATGTTTTCT-3′ were used to amplify exons 9 and 10, respectively. Exon 9 was sequenced using the forward primer 5′-GCTGTTGTCATCTCAGTGAATTC-3′ and the reverse primer 5′-CCACAACTTCACTATCATCCAG-3′. Exon 10 was sequenced using the forward primer 5′-CCAATTACTGTTGTCATCACAG-3′ and the reverse primer 5′-TAGACTGTCCTCCCAGCAACTC-3′. Multiple sequence alignments were performed using Clustal Omega (50).

### BrdU incorporation assay

DNA synthesis was assessed in THP-1 cells using previously described methods. Briefly, THP-1 cells were stimulated with PMA for 7 days or grown in suspension culture. Media was replaced with complete RPMI containing 10 μm 5-bromo-2′-deoxyuridine (Sigma). Cells were grown in the presence of BrdU for 1 or 8 h, harvested, and fixed by addition of 70% ice-cold ethanol. Cells were permeabilized by addition of 0.5% Triton X-100 and DNA was denatured with 2 n HCl, neutralized, washed, and stained with an anti-BrdU antibody (Cell Signaling Technology; Bu20a) diluted 1:50 in a solution of 1× PBS, 1% BSA, and 0.1% Tween 20. Cells were washed then stained with Alexa Fluor 488 goat anti-mouse antibody (Invitrogen) diluted 1:50. Cells were washed and analyzed using a MACSQuantVYB flow cytometer (Miltenyi Biotec) and data were analyzed using MACSQuantify software (Miltenyi Biotec).

### Western blotting

Anti-β-actin (Abcam; ab6276) and anti-Pol β (Abcam; ab175197) antibodies were used for Western blotting. Anti-Pol β antibodies were first validated for Western blotting using nuclear and whole cell extracts from THP-1, Jurkat, HeLa, and 293FT cells. Lysates were resolved by SDS-PAGE on a 4–15% gel (Bio-Rad) and proteins were transferred to a nitrocellulose membrane (Bio-Rad). Primary antibodies were diluted 1:5,000 in TBS-T with 5% dry milk. Anti-species secondary antibodies were diluted 1:10,000 in TBS-T with 5% dry milk. Blots were imaged by chemiluminescence (SuperSignal West Femto maximum sensitivity substrate, Thermo Scientific) using a ChemiDoc Touch imaging system (Bio-Rad) and analyzed in ImageLab 5.2 software (Bio-Rad). A specific band at ∼38 kDa corresponding to the predicted molecular weight for Pol β was detected in all samples tested with slightly varying levels of expression depending on cell type.

### MMS sensitivity assay

Sensitivity to the DNA damaging agent MMS (17) was determined by measuring growth inhibition using the tetrazolium salt-based XTT assay (ATCC) (51). Assay linearity was predetermined by varying cell seeding density, incubating cells at 37 °C, 5% CO_2_ for 72 h, and measuring specific absorbance (475 nm- 660 nm) using an Epoch microplate spectrophotometer (BioTek) after a 4-h incubation with XTT reagent. For evaluation of MMS cytotoxicity, cells were incubated with varying concentrations of MMS (Sigma) for 2 h, washed 3 times with 1× PBS, and incubated at 37 °C, 5% CO_2_ for 72 h before being assayed as described above.

### Preparation of nuclear extracts

Nuclear extracts were prepared as previously described (52). Briefly, cells were washed in 1× PBS, resuspended in hypotonic buffer, and Dounce homogenized. Lysed cells were centrifuged at 3,300 × *g* for 15 min. The nuclear pellet was resuspended in low salt extraction buffer and mixed dropwise with high salt buffer (1.6 m KCl). Extracted nuclei were centrifuged at 22,065 × *g* for 30 min. Supernatant was collected and centrifuged for an additional 15 min at 22,065 × *g*. Extracts were dialyzed against storage buffer for 2 h, aliquotted, and stored at −80 °C. Total protein concentration was determined by the Bradford assay (Bio-Rad).

### In vitro gap repair assay

*In vitro* gap repair assays were performed as previously described (11) using previously published oligonucleotides (4). The primers KEY35 (5′-ATTCGAGCTATCCTTGCGCG-3′) and KEY31 (5′-ACTGCTAGAGATTTTCCACACTGACTA-3′) and the template KEY36 (5′-TAGTCAGTGTGGAAAATCTCTAGCAGGCCCCGCGCAAGGATAGCTCGAAT-3′) were obtained from Integrated DNA Technologies. KEY35 was 5′ end-labeled with γ-^32^P using T4 polynucleotide kinase (New England Biolabs). Gap repair substrate was made by annealing 800 nm KEY35, 2.4 μm KEY31, and 1.6 μm KEY36.

Gap repair reactions were performed in 20-μl volumes containing 20 nm substrate, 2 mm ATP, dNTPs (60 nm, 2.5 μm, or 250 μm), and reaction buffer. Reactions were started by adding 4 μl of 1 mg/ml of nuclear extract, incubated at 37 °C for 30, 60, or 120 min, then stopped by addition of 10 μl of 40 mm EDTA, 99% formamide and heated for 1 min at 95 °C. A 4-μl aliquot from each reaction was resolved by 20% urea-PAGE (American Bio). Gels were visualized by phosphorimaging on a PharoxFX Molecular Imager (Bio-Rad) and quantitated using ImageLab 5.2 software (Bio-Rad).

## Author contributions

R. W. G. designed and performed the experiments, analyzed the data, and wrote the paper. R. F. S. and D. H. K. conceived the research. B. K. conceived the research, designed the experiments, and wrote the paper. All authors read and approved the final manuscript.

## Supplementary Material

Supplemental Data

## Footnotes

2 The abbreviations used are:

SIV: simian immunodeficiency virus
IN: integrase
BER: base excision repair
sgRNA: single guide RNA
PMA: phorbol 12-myristate 13-acetate
MMS: methyl methanesulfonate
VSV: vesicular stomatitis virus
XTT: 2,3-Bis-(2-Methoxy-4-Nitro-5-Sulfophenyl)-2*H*-Tetrazolium-5-Carboxanilide.
