# Supplementary material for: A CRISPR/Cas9 approach reveals that the polymerase activity of DNA polymerase β is dispensable for HIV-1 infection in dividing and nondividing cells
Source: J Biol Chem. 2017 Jul 6;292(34):14016–25. doi: 10.1074/jbc.M117.793661 (PMC5572920; doi:10.1074/jbc.M117.793661)
Supplement: Supplemental Data [file supp_292_34_14016__index.html]

A CRISPR/Cas9 approach reveals that the polymerase activity of DNA Polymerase β is dispensable for HIV-1 infection in dividing and nondividing cells — A CRISPR/Cas9 approach reveals that the polymerase activity of DNA polymerase β is dispensable for HIV-1 infection in dividing and nondividing cells — Host DNA Pol β and HIV-1 gap repair — Supplemental Data 

# A CRISPR/Cas9 approach reveals that the polymerase activity of DNA polymerase β is dispensable for HIV-1 infection in dividing and nondividing cells

## Supplemental Data

- Supplemental Figure 1 (.docx, 132 KB) - Supplemental Figure 1
- Supplemental Figure 2 (.docx, 2.3 MB) - Supplemental Figure 2
