# Supplementary material for: A CRISPR/Cas9 approach reveals that the polymerase activity of DNA polymerase β is dispensable for HIV-1 infection in dividing and nondividing cells
Source: J Biol Chem. 2017 Jul 6;292(34):14016–25. doi: 10.1074/jbc.M117.793661 (PMC5572920; doi:10.1074/jbc.M117.793661)
Supplement: Supplemental Data [file 10.1074_M117.793661_jbc.M117.793661-1.docx]

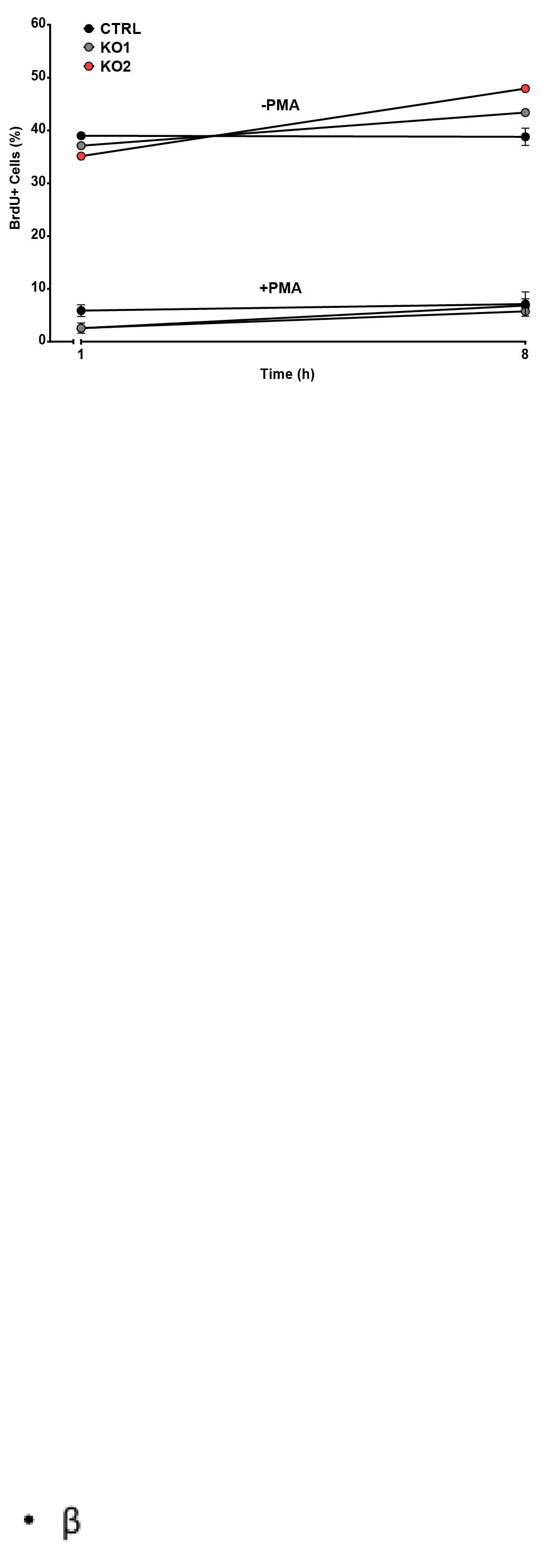


**Figure S1: PMA-treated THP-1 cells have minimal levels of DNA synthesis.** DNA synthesis was measured in the dividing and non-dividing stages of THP-1 CTRL, KO1, and KO2 cells using BrdU incorporation. Cells were cultured in complete media containing 10 μM BrdU for 1 or 8 h. Approximately 40% of dividing stage cells were positive for BrdU incorporation after 8 h treatment for all three cell lines. In contrast, PMA-treated cells in the non-dividing/macrophage stage showed only up to 8% of cells as positive after 8 h treatment, indicating only a low level of background DNA synthesis in these cells. Data are shown as mean ± S.D. of two independent experiments performed in triplicate.
