# Supplementary material for: A CRISPR/Cas9 approach reveals that the polymerase activity of DNA polymerase β is dispensable for HIV-1 infection in dividing and nondividing cells
Source: J Biol Chem. 2017 Jul 6;292(34):14016–25. doi: 10.1074/jbc.M117.793661 (PMC5572920; doi:10.1074/jbc.M117.793661)
Supplement: Supplemental Data [file 10.1074_M117.793661_jbc.M117.793661-2.docx]

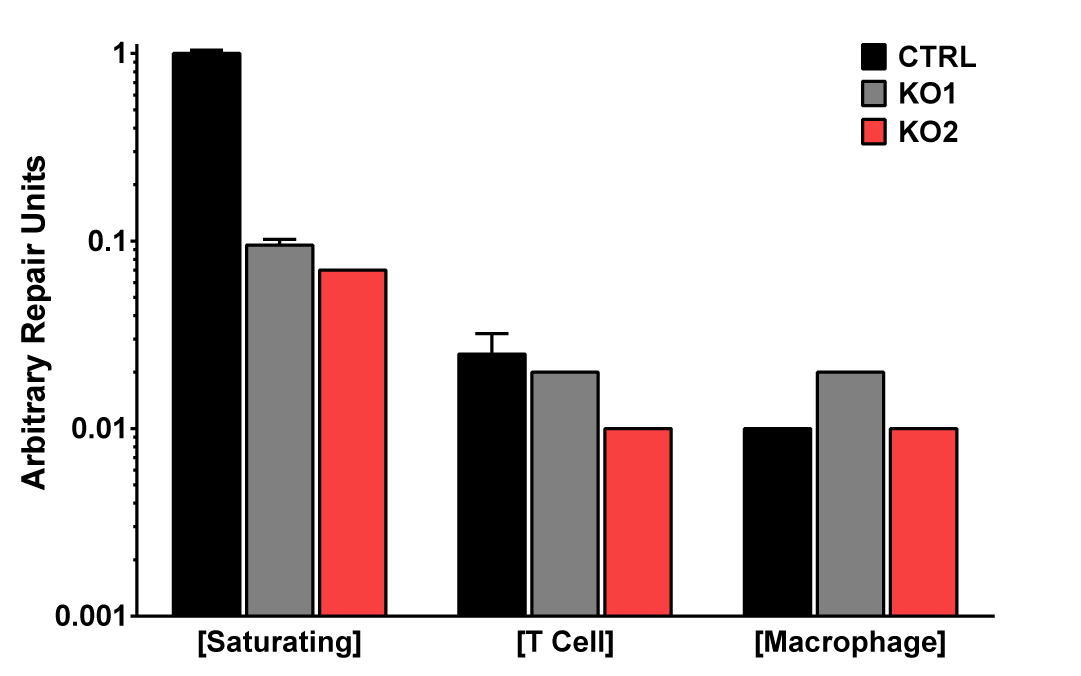


**Figure S2: Effect of dNTP concentration on *in vitro* HIV-1 ssDNA gap repair activity in *POLB* KO THP-1 cells.** Nuclear extracts from dividing (-PMA) and nondividing (+PMA) stages of THP-1 CTRL, KO1, and KO2 cells were isolated. 20 nM gap repair substrate was incubated with 4 μg nuclear extract in the presence of saturating (250 μM), dividing cell (5 μM), or nondividing cell (40 nM) concentrations of dNTPs and 2 mM ATP (required for ligation). Reactions were incubated for 120 min. Data from two independent experiments were quantitated by densitometry to quantitate the amounts of fully repaired substrate which are reported as mean ± S.D for dividing stages of THP-1 cells.
